# Presence of factors that activate platelet aggregation in mitral stenotic patients' plasma

**DOI:** 10.1186/1468-6708-6-2

**Published:** 2005-02-27

**Authors:** Istemihan Tengiz, Ertugrul Ercan, Fahri Sahin, Emin Alioglu, Can Duman, Guray Saydam, Filiz Buyukkececi

**Affiliations:** 1 Department of Cardiology, Central Hospital, 1644 sk. No:2/2, 35010 Bayrakli, Izmir, Turkey; 2 Department of Hematology, Ege University Medical School, 35100 Bornova, Izmir, Turkey; 3 Department of Biochemistry, Kocaeli University Medical School, 41900 Derince, Kocaeli, Turkey

## Abstract

**Background:**

Although the association between mitral stenosis (MS) and increased coagulation activity is well recognized, it is unclear whether enhanced coagulation remains localized in the left atrium or whether this represents a systemic problem. To assess systemic coagulation parameters and changes in platelet aggregation, we measured fibrinogen levels and performed *in vitro *platelet function tests in plasma obtained from mitral stenotic patients' and from healthy control subjects' peripheral venous blood.

**Methods:**

Sixteen newly diagnosed patients with rheumatic MS (Group P) and 16 healthy subjects (Group N) were enrolled in the study. Platelet-equalized plasma samples were evaluated to determine *in vitro *platelet function, using adenosine diphosphate (ADP), collagen and epinephrine in an automated aggregometer. *In vitro *platelet function tests in group N were performed twice, with and without plasma obtained from group P.

**Results:**

There were no significant differences between the groups with respect to demographic variables. Peripheral venous fibrinogen levels in Group P were not significantly different from those in Group N. Adenosine diphosphate, epinephrine and collagen-induced platelet aggregation ratios were significantly higher in Group P than in Group N. When plasma obtained from Group P was added to Group N subjects' platelets, ADP and collagen-induced, but not epinephrine-induced, aggregation ratios were significantly increased compared to baseline levels in Group N.

**Conclusion:**

Platelet aggregation is increased in patients with MS, while fibrinogen levels remain similar to controls. We conclude that mitral stenotic patients exhibit increased systemic coagulation activity and that plasma extracted from these patients may contain some transferable factors that activate platelet aggregation.

## Introduction

Systemic thromboembolism represents a major complication in patients with mitral stenosis (MS), especially in those who have atrial fibrillation [1,2]. A hypercoagulable state has been reported in patients with MS and sinus rhythm [3,4]. The association between MS and higher levels of coagulation is well-known; however, the source of increased coagulation remains unclear. Increased regional left atrial coagulation activity may be involved, even when systemic coagulation assessed by peripheral blood sampling is normal [5,6]. Others have reported no significant variation in thrombogenesis, platelet activation, and endothelial dysfunction between the left atrium, right atrium and peripheral arteries or veins [7].

This study assessed systemic coagulation and platelet activities by measuring fibrinogen levels and platelet aggregation in mitral stenotic patients' peripheral venous blood and compared these measures to hematologic parameters in normal controls.

## Methods

### Study groups

Eighteen newly diagnosed patients with rheumatic MS were enrolled in the study. All were coded as having NYHA class I functional capacities. Two patients were excluded from this study, one due to pregnancy, the second due to current use of oral contraceptives. The study group thus consisted of 16 patients with MS (Group P, 14 female, 2 male, mean age 27.8 ± 6.5 years). Peripheral venous coagulation activity and platelet activation tests were also evaluated in 16 control patients (Group N, 13 female, 3 male, mean age 28.3 ± 6.1 years), who were normal volunteers. All participants gave informed consent.

### Exclusion criteria

Patients were excluded from participating in the study for the following reasons: aortic and/or pulmonary valve disease, severe mitral regurgitation and/or left ventricular systolic dysfunction, atrial fibrillation, hypertension, diabetes mellitus, dyslipidemia, a history of renal or liver disease, malignancy, venous thrombosis, systemic or pulmonary embolism, congenital hemorrhagic disease, thrombocytopenia, thrombocytosis, acute or chronic inflammatory disease, autoimmune disease or current use of oral contraceptives or anticoagulant or anti-platelet drugs.

### Echocardiography

In patients with MS, transthoracic echocardiography was performed with a 2.5-MHz transducer and a Hewlett Packard Sonos 4500 system to assess left atrial (LA) diameter, mitral valve area (MVA) and the transmitral mean pressure gradient (TMmPG). The LA anteroposterior diameter was determined using standard M-mode criteria [8], and mitral valve area was calculated according to the pressure half-time method [9].

### Fibrinogen measurement

Peripheral venous blood (for measurement of fibrinogen levels) was transferred into tubes containing 1:9 trisodium citrate (0.109 M, 3.2%). Blood samples were centrifuged within 1 hour of collection at 2,500 g for 10 minutes to separate out the plasma. An STA fibrinogen kit measured fibrinogen levels in plasma, using the clotting method of Clauss. The median inter-assay and intra-assay coefficients of variation for the assays were 3.57% and 3.65% for fibrinogen, respectively.

### Platelet activation tests

Blood samples were obtained from the forearm veins of participants, using standard blood drawing procedures (normal blood flow and no pressure). Several samples were drawn for different components of the study. Nine milliliters of blood was collected into special tubes containing 1 ml of 3.8% sodium citrate. Platelet count and other parameters, including white blood cell count and hemoglobin, were measured at the Ege University Hospital Hematology Laboratory using an automated hemocytometer (Cell Dyne 4000 Abbott, USA). Patients with platelet counts less than 2 × 10^5^/ml were excluded from the study. For *in vitro *platelet aggregation tests, platelet-rich plasma (PRP) samples were obtained by centrifuging samples at 1,100 rpm for 10 minutes in a refrigerated centrifuge (Hettich EBA 12R, Germany). Centrifuging samples at 3,000 rpm for 10 minutes yielded platelet-poor plasma (PPP) samples. The platelet counts of both PRP and PPP samples were performed using the same hemocytometer. Platelet-poor plasma samples were added to the PRP samples to obtain a total platelet count of 2 × 10^5^/ml. These platelet-equalized plasma (PEP) samples were analyzed to determine *in vitro *platelet function, using ADP, Collagen and Epinephrine (all from Bio/Data Corp. Horshram, Germany) in an automated aggregometer (Bio/Data Corp. Platelet Agregation Profiler, Horshram-Germany). Results were evaluated and calculated using maximum aggregation ratios.

PRP from healthy controls was centrifuged for 10 minutes, after which the supernatant was decanted, and normal saline added to the platelet fraction and centrifuged for 10 minutes. This procedure was repeated three times. Plasma obtained from patients with MS was added to the PPP from the control group, and platelet function tests were performed, using the same procedures mentioned above.

### Statistical analyses

Chi Square tests were employed to compare categorical variables, while Mann Whitney U tests were used, where appropriate, in the univariate analysis. Platelet aggregation changes were evaluated by repeated-measures analysis of variance for intra-group comparisons. Values of p < 0.05 were considered significant. SPSS 10.0 Statistical software was used in the statistical analysis.

## Results

There were no significant differences between Groups P and N for the following variables: age, gender, current smoker, platelet, white blood cell and hemoglobin count. Clinical characteristics of the study groups are shown in Table 1. The peripheral venous fibrinogen levels in patients with MS (301.9 ± 53.7 mg/dL) were not significantly different from those in control subjects (284.6 ± 55.7 mg/dL).

**Table 1:** Clinical features of the study groups and echocardiographic findings of patients with mitral stenosis.

	**Group P (n = 16)**	**Group N (n = 16)**	**p**
**Mean age (yrs)**	27.8 ± 6.5	28.3 ± 6.1	NS
**Male/female (n)**	2/14	3/13	NS
**Current smoker (n)**	5	6	NS
**Platelet count × 10^3^/mm^3^**	253.1 ± 33.5	245.6 ± 34.3	NS
**WBC/mm^3^**	5706 ± 1266	5643 ± 1229	NS
**Hb (g/L)**	12.4 ± 1.26	12.8 ± 1.22	NS
**Fibrinogen (mg/dL)**	301.9 ± 53.7	284.6 ± 55.7	NS
**LA diameter (cm)**	4.22 ± 0.19	-	-
**MVA (cm^2^)**	1.67 ± 0.16	-	-
**TMmPG (mmHg)**	6.5 ± 0.96	-	-
**Mitral regurgitation (n)**			
None	4	-	-
Mild	11	-	-
Moderate	1	-	-

**Group P: **Patients with mitral stenosis, **Group N: **Control subjects, **WBC: **White blood cell, **Hb: **Hemoglobin, **LA: **Left atrium, **MVA: **Mitral valve area, **TMmPG: **Transmitral mean pressure gradient, **NS: **Non-significant.

### Platelet activation tests

**Figure 1:**
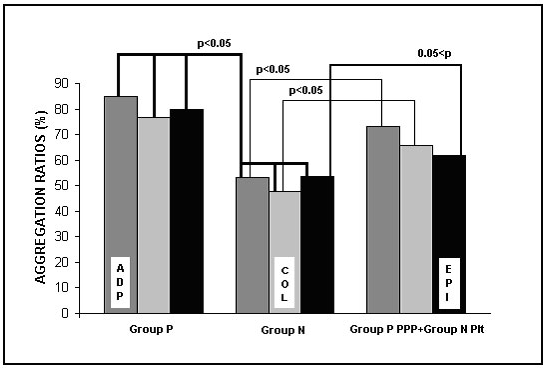
Induced platelet aggregation ratios in Groups P and N. Group P: Patients with mitral stenosis, Group N: Control subjects, PPP: Platelet-poor plasma, Plt: platelet, ADP: Adenosine diphosphate, COL: Collagen, EPI: Epinephrine.

Adenosine diphosphate, collagen and epinephrine-induced platelet aggregation ratios were significantly higher in Group P than in Group N (84.9 ± 9.8, 76.6 ± 21.4, 80 ± 10.5 and 53.1 ± 23.2, 47.5 ± 14.5, 53.7 ± 21.3, p < 0.05, respectively). When the PPP obtained from Group P was added to Group N subjects' platelets, and platelet function tests were repeated, ADP and collagen-induced (73 ± 17.5 and 65.7 ± 17.5, respectively), but not epinephrine-induced (61.9 ± 16.2), aggregation ratios were statistically increased compared to baseline (Figure 1).

## Discussion

Left atrial thrombus in MS is associated with increased systemic levels of peptide by-products from the coagulation cascade [10,11]. Increases in markers of coagulation activity may also occur in the absence of thrombus, perhaps signifying that an imbalance in hemostatic regulation favoring pro-coagulant mechanisms may precede and predispose to thrombus formation [12,13]. Information about coagulation activity in patients with MS could, therefore, provide a method of identifying patients at risk of developing systemic thromboembolism.

A number of studies have reported that indexes of hypercoagulation derived from peripheral blood may not reflect intracardiac thrombogenesis. Yamamoto et al. [6] noted that fibrinopeptide A and thrombin/antithrombin III were higher in the left atrium compared to the right atrium and the femoral vein. Peverill et al. [5] found that levels of prothrombin fragments 1+2 increased in the left atrium in comparison to peripheral blood levels. Li-Saw-Hee et al. [7] reported no significant variation in indexes of thrombogenesis, platelet activation, and endothelial dysfunction between left atrium, right atrium, and the peripheral artery or vein. A number of differences between these studies and our study may, in part, account for these varying observations. All of our patients exhibited newly diagnosed, mild-to- moderate MS, and none was taking anticoagulants or anti-platelet drugs. Second, we did not investigate systemic levels of peptide by-products of the coagulation cascade or individual blood components that reflect coagulability. In our experimental design, we sought to examine the effect of mitral stenotic patients' plasma on control subjects' platelets.

In addition to protein factors, platelets play a vital role as systemic components of the hemostatic system. They are critical to the activation of intrinsic pathway factors. Because platelet activation seems to be influenced by diabetes mellitus [14], smoking [15], hypertension and the use of oral contraceptives [16], patients with these conditions were excluded from our study.

Hwang et al. [17] sought to examine a correlation among echocardiographic variables, hematologic parameters or platelet aggregability and the occurrence of spontaneous echo-contrast (SEC) in the left atrium. Platelet aggregability was evaluated with a turbidometric method, using different concentrations of activating agents. No significant difference was found in platelet aggregability between patients with left atrial SEC and patients without left atrial SEC. Neither group of patients had been receiving anti-platelet or anticoagulant therapy.

Ileri et al. [4] observed hypercoagulation in patients with mitral stenosis and sinus rhythm when SEC was present. Pongratz et al. [18] investigated the activation status of platelets in the peripheral blood of patients with atrial fibrillation. A significantly higher level of circulating platelets expressing P-selectin and CD63 and more leukocyte-platelet conjugates were found in patients positive for both SEC and left atrial thrombus or embolic events. Increased spontaneous platelet aggregation in the presence of SEC was described by Rohmann et al. [19], who measured the half-maximal formation of platelet aggregates in peripheral blood, upon stimulation with ADP.

In our study, all patients had mild-to-moderate MS, with functional capacities rated as being NYHA class I. Although peripheral fibrinogen levels were similar between Group P and N, platelet aggregation was significantly higher in Group P than in Group N. Moreover, alteration of platelet aggregation in Group N, triggered by the addition of plasma from mitral stenotic patients, may indicate the presence of possible transferable platelet activators in peripheral blood.

In conclusion, we examined systemic coagulation activity in patients with MS by measuring plasma level of fibrinogen and by performing platelet activation tests in peripheral venous blood samples. Our examinations yielded three main findings. First, mitral stenotic patients have similar fibrinogen levels in peripheral venous blood as compared to controls. Second, platelet aggregation is higher in patients with MS than in controls. Third, in comparison to controls, patients with MS have increased systemic coagulation activity and the plasma extracted from these patients seems to have some transferable factors that activate platelets.

The results from this study emphasize the complexity of the events related to platelet activation in MS. Also compounding the challenges faced in this area is uncertainty about the gold standard method for assessing platelet activation and whether platelet aggregation (*ex vivo*) truly reflects platelet activation *in vivo *[20].
